# The crescent-like Golgi ribbon is shaped by the Ajuba/PRMT5/Aurora-A complex-modified HURP

**DOI:** 10.1186/s12964-023-01167-4

**Published:** 2023-06-27

**Authors:** Shao-Chih Chiu, Xin-Ting Yang, Tong-You Wade Wei, Yu-Ting Amber Liao, Jo-Mei Maureen Chen, Yi-Chun Kuo, Chun-Chih Jared Liu, Chiao-Yun Cheng, Yu-Ting Jenny Huang, Yun-Ru Jaoying Huang, He-Lian Joe Wu, Chang-Xin Wan, Jia-Rung Tsai, Chang-Tze Ricky Yu

**Affiliations:** 1 Department of Medical Research, Translational Cell Therapy Center, China Medical University Hospital, Taichung, Taiwan; 2 Graduate Institute of Biomedical Sciences, China Medical University, Taichung, Taiwan; 3 Department of Applied Chemistry, National Chi-Nan University, Nantou, Taiwan; 4 Graduate Institute of Biomedicine and Biomedical Technology, National Chi Nan University, Nantou, Taiwan; 5 Department of Medicine, University of California, San Diego, CA USA; 6 Division of Hematology/Medical Oncology, Department of Medicine, Taichung Veterans General Hospital, Taichung, Taiwan; 7 Present Address: Department of Applied Chemistry, National Chi Nan University, No. 1, University Rd. Puli, Nantou, 545 Taiwan

**Keywords:** Golgi ribbon, HURP, Ajuba, PRMT5, Aurora-A, ARF1

## Abstract

**Background:**

Golgi apparatus (GA) is assembled as a crescent-like ribbon in mammalian cells under immunofluorescence microscope without knowing the shaping mechanisms. It is estimated that roughly 1/5 of the genes encoding kinases or phosphatases in human genome participate in the assembly of Golgi ribbon, reflecting protein modifications play major roles in building Golgi ribbon.

**Methods:**

To explore how Golgi ribbon is shaped as a crescent-like structure under the guidance of protein modifications, we identified a protein complex containing the scaffold proteins Ajuba, two known GA regulators including the protein kinase Aurora-A and the protein arginine methyltransferase PRMT5, and the common substrate of Aurora-A and PRMT5, HURP. Mutual modifications and activation of PRMT5 and Aurora-A in the complex leads to methylation and in turn phosphorylation of HURP, thereby producing HURP p725. The HURP p725 localizes to GA vicinity and its distribution pattern looks like GA morphology. Correlation study of the HURP p725 statuses and GA structure, site-directed mutagenesis and knockdown-rescue experiments were employed to identify the modified HURP as a key regulator assembling GA as a crescent ribbon.

**Results:**

The cells containing no or extended distribution of HURP p725 have dispersed GA membranes or longer GA. Knockdown of HURP fragmentized GA and HURP wild type could, while its phosphorylation deficiency mutant 725A could not, restore crescent Golgi ribbon in HURP depleted cells, collectively indicating a crescent GA-constructing activity of HURP p725. HURP p725 is transported, by GA membrane-associated ARF1, Dynein and its cargo adaptor Golgin-160, to cell center where HURP p725 forms crescent fibers, binds and stabilizes Golgi assembly factors (GAFs) including TRIP11, GRASP65 and GM130, thereby dictating the formation of crescent Golgi ribbon at nuclear periphery.

**Conclusions:**

The Ajuba/PRMT5/Aurora-A complex integrates the signals of protein methylation and phosphorylation to HURP, and the HURP p725 organizes GA by stabilizing and recruiting GAFs to its crescent-like structure, therefore shaping GA as a crescent ribbon. Therefore, the HURP p725 fiber serves a template to construct GA according to its shape.

Video Abstract

**Supplementary Information:**

The online version contains supplementary material available at 10.1186/s12964-023-01167-4.

## Background

The Golgi apparatus (GA) in mammalian cells forms a continuous ribbon of laterally interconnected stacks of flat cisternae. The construction of GA architecture relies on posttranslational modifications of soluble Golgi assembly factors (GAFs) and is subjected to dynamic changes along a cell cycle [2]. At G2 phase, phosphorylation-induced inactivation of GRASP65 [7] or GRASP55 [15], two GAFs engaged in stacking and linking cis- and trans-Golgi respectively, leads to the unlinking of the inter-connected stacks of the organelle. At early mitosis, further phosphorylation of the two GRASPs causes GA unstacking [41, 45]. Subsequently, phosphorylation of GBF1, the activator of ARF1, dissociates GBF1 from Golgi membrane and inactivates ARF1, further breaking GA into Golgi blobs or hazes throughout mitotic cytoplasm [1, 28, 31]. ARF1 is a key GAF recruiting a range of downstream GAFs to GA, thereby initiating GA assembly process [23]. For example, ARF1 induces a constant centripetal transport of Golgi membranes to assemble GA at the cell center by attracting Golgin-160, a Dynein cargo adaptor engaged in transporting Golgi membranes [46]. TRIP11, mustered by ARF1, participates in asymmetric tethering of flat and curved lipid membranes [14] and homotypic fusion of cis-cisternae [6]. In addition to ARF1-dependent pathways, some GAFs such as GM130 are recruited to GA in an ARF1 independent manner [17]. GM130, p115 and Giantin form a complex that captures COPI vesicles on the cis-Golgi for future fusion [40], and phosphorylation of p115 is required for Golgi assembly [12]. The final stage of Golgi assembly is accomplished by the linking of Golgi stacks, thereby lengthening GA into an extended ribbon. Many factors are involved in the linking process such as TRIP11 [35], GRASP65 and GM130 [34], and methylation of GM130 by PRMT5 is important for Golgi ribbon construction [49]. A study points out that 159 genes, nearly 20% of the genes assayed, in the human genome encoding kinases or phosphatases participate in the regulation of GA architecture [10], which reflects GA assembly is regulated by a huge number of protein modifications. However, most of such modifications on GAFs, such as phosphorylation or methylation, remain unidentified. Intriguingly, the lengthened Golgi ribbon displays a bent architecture and looks like a crescent under the fluorescence microscope. How the Golgi membranes are assembled as a crescent-like ribbon with a curved architecture is completely unknown.

To explore how the GA is shaped as a crescent organelle under the guidance of protein modifications, we firstly identify a protein complex which is organized by a scaffold protein Ajuba [29] and contains two reported GA regulators, i.e. arginine methyltransferase PRMT5 [49] and serine/threonine kinase Aurora-A [25], and their common substrate HURP [11, 48]. HURP is a versatile factor regulating spindle stability [39] and chromosome congression [47], promoting G1/S cell cycle progression [8], and inhibiting apoptosis [18]. Mutual modification and subsequent activation of PRMT5 and Aurora-A in the protein complex catalyzes the methylation and in turn phosphorylation of HURP. HURP p725, i.e. the HURP with phosphorylation at S725, is then transported by ARF1/Golgin-160/Dynein to the cell center, where HURP p725 displays a crescent-like structure, binding and stabilizing GAFs such as TRIP11, GRASP65 and GM130, thereby facilitating the assembly of the bulky GA along the strong, crescent HURP p725 structure.

## Methods

### Antibodies, shRNA, plasmids, and reagents

The following antibodies were used in the study: alpha tubulin (sc-5286), Golgin-160 (sc79966), actin (sc-8432), PRMT5 (sc-22132), Ajuba (sc-374610), GP73 (48010), and ARF1 (sc053168) were purchased from Santa cruz; GRASP65 (ab174834), Aurora-A (ab1287), Aurora-A p288 (ab83968), and GBF1 (ab86071) were from Abcam; c-Myc (M4439), HA (H3663) and FLAG (F7425) were from Sigma; GM130 (H00002801-B01P) was from Abnova; TRIP11 (MA 1–23294) and Dynein (MA 1–070) were from Thermo; GFP (11814460001) from Roche; GRASP55 (10598–1-AP) from Proteintech; ERGIC3 (CSB-PA896688LA01HU) from Cusabio. The shRNA clones carried in Lentivirus backbone were from National RNAi Core Facility at Academia Sinica in Taiwan with the following clone number and target sequence: Luciferase, GCGGTTGCCAAGAGGTTCCAT; HURP, GCACAGCAGTTGGTCAAACAA; PRMT5, GCCCAGTTTGAGATGCCTTAT; Ajuba, GCTCCTTATCTGTCTGAGAAT; TRIP11, GCAAAGGAACAAGAACTCAAT; GRASP65, CGAGGACTTCTTTACGCTCAT; Aurora-A, CCTGTCTTACTGTCATTCGAA; ARF1, AGAAATTGGAGAAAGTTAAAG; GBF1, CACGACACTAAGTCTCTGCTT; Golgin-160, GCAGAACGTCAAGTCTGAGTT. The expression plasmids used in the study were from different donors including Myc-Ajuba from Dr. Hirota [19], Myc-GRASP65 from Dr. Feng [16], EGFP-Golgin-160 from Dr. Maag [27], GFP-TRIP11 from Dr. Lee [9], and HA-ARF1 WT and T31N were from addgene. EGFP-HURP R122K and 122F were from our previous study [11], HA-HURP 725A and 725E were from our previous study [42], The key chemicals such as BFA (Brefeldin A), T3 (3’,5-Triiodo-L-thyronine sodium salt), and cycloheximide were from Sigma-Aldrich; Ciliobrevin D from MERCK.

### Preparation of antibodies against methyl- or phospho-antibodies

The antibodies against Aurora-A m304 or nm304, PRMT5 p103 or np103, HURP m122 or nm122, HURP p725 or np725, were generated by immunizing rabbits or mice with commercially synthesized KLH-linked peptides containing methyl-R or phospho-S/T at the center of the following sequences: Aurora-A m304, PPEMIEG(mR)MHDEKVD; PRMT5 p103, VEKIRRN(pS)EAAML; HURP m122, GIFKVG(mR)YRPDMP; HURP p725, LSSERM(pS)LPLLA. The peptides were conjugated to a KLH-hapten carrier protein to generate significant immune response. The corresponding unmodified peptide counterparts with same sequences were also synthesized for affinity purification and subsequent validation. The elicited antibodies were affinity-purified from the antisera by columns packed with the same peptides used for immunization through two processes. Firstly, the antisera were applied to the unmodified peptides-packed column, and the flow-through was subjected to the second column packed with modified peptide. The absorbed antibodies in the second column were eluted and considered as antibodies recognizing modified peptide. Alternatively, the absorbed antibodies in the first column were eluted and applied to the modified peptide-packed column, and the resulted flow-through was the antibodies recognizing unmodified peptide. Finally, dot blot and Western blot were adopted to validate the antibodies.

### Site-directed mutagenesis

The phosphorylation or methylation mutants employed in the study, including EGFP-PRMT5 S103A, FLAG-Aurora-A R304K, EGFP-HURP R122K, EGFP-HURP S725A and S725E, EGFP-TRIP11, were generated using PCR-based mutagenesis (QuickChange Site-Directed Mutagenesis Kit, Agilent Technologies) according to manufactory’s instruction. The employed primer sequences were listed in the followings: HURP R122K (forward), GGA ATA TTT AAA GTG GGT AAG TAT AGA CCT GAT ATG CC, HURP R122K (reversed), GG CAT ATC AGG TCT ATA CTT ACC CAC TTT AAA TAT TCC; HURP R122F (forward), GGA ATA TTT AAA GTG GGT TTT TAT AGA CCT GAT ATG CC, HURP R122F (reversed), GG CAT ATC AGG TCT ATA AAA ACC CAC TTT AAA TAT TCC; HURP S725A (forward), TTT ATC CAG TGA GAG AAT GGC TTT GCC TCT TCT TGC TGG TG, HURP S725A (reversed), CAC CAG CAA GAA GAG GCA AAG CCA TTC TCT CAC TGG ATA AA; HURP S725E (forward), TTG TTT ATC CAG TGA GAG AAT GGA GTT GCC TCT TCT TGC TGG TGG AG, HURP S725E (reversed), CTC CAC CAG CAA GAA GAG GCA ACT CCA TTC TCT CAC TGG ATA AAC AA; Aurora-A R304K (forward), G CCC CCT GAA ATG ATT GAA GGT TTT ATG CAT GAT GAG AAG GTG GAT C, Aurora-A R304F (reversed), G ATC CAC CTT CTC ATC ATG CAT AAA ACC TTC AAT CAT TTC AGG GGG C; PRMT5 S103A (forward), GAT TCG CAG GAA CGC CGA GGC GGC CAT, PRMT5 S103A (reversed), ATG GCC GCC TCG GCG TTC CTG CGA ATC.

### Cell lines and cell cultures

The 293 (HEK293), 293T and HeLa (ATCC CRL-1573, CRL-3216 and CCL-2 respectively) were maintained in a humidified incubator at 37 ˚C in the presence of 5% CO_2_, and were grown in a DMEM medium containing 5% FBS, 100 unit/mL penicillin and 100 μg/mL streptomycin.

### Cell Cultures, transfection and lentiviral-based RNA interference

The 293, 293T and HeLa were maintained in a humidified incubator at 37 ˚C in the presence of 5% CO_2,_ and were grown in a DMEM medium containing 5% FBS, 100 unit/mL penicillin and 100 μg/mL streptomycin. Transfection was performed with Lipofectamine™ 2000 (Life Technologies) or Polyjet™ (SignaGen Laboratories) according to the manufacturer’s instructions. The shRNAs carried by lentiviral backbones were obtained from the National RNAi core facility (Institute of Molecular Biology, Academia Sinica, Taiwan) with the targeting sequences listed in key resources table.

### Preparation of cell extracts, Western blot, and immunoprecipitation

The cell extracts were prepared using an extraction buffer consisting of 50 mM Tris pH7.5, 0.1% SDS, 1% NP40, 0.5% sodium deoxycholate, 1% Triton X-100, 5 mM EDTA, 150 mM NaCl, and 150 mM KCl. Protein concentration was determined by the Bradford assay (Bio-Rad). Equal amounts of total lysates were used for further analyses, or loaded onto a 10% SDS–polyacrylamide electrophoresis gel (SDS-PAGE) and transferred onto a PVDF membrane (Amersham). The PVDF membranes were blocked with 5% skimmed milk/TBST (150 mM Sodium Chloride, 20 mM Tris, 0.1% Tween-20, pH 7.6). Primary antibodies were incubated with the membranes at 4 °C for 2 h. The membranes were washed with TBST for 30 min and this was repeated 3 times. Secondary antibodies, conjugated with alkaline phosphatase (AP, Santa cruz) or horseradish peroxidase (HRP, AffiniPure, Jackson ImmunoResearch), were added for 1 h, followed by washing with TBST for 3 × 30 min. AP’s substrate BCIP/NBT (Renaissance, PerkinElmer), or HRP’s substrate (WesternBright, Advansta) were added to develop the membranes. As to immunoprecipitation, 1–2 mg of cell extracts with protease inhibitor cocktail (Roche) were incubated with protein A/G beads (Roche) in 500 μl immunoprecipitation washing buffer (50 mM HEPES, pH 7.6, 2 mM MgCl2, 50 mM NaCl, 5 mM EGTA, 0.1% Triton X-100, 40 mM glycerolphosphate) at 4 °C for 1 h to preabsorb unwanted proteins. 1 μg antibody were then added to the cell extracts for 4 h at 4 °C. The cell extracts were incubated with protein A/G-beads for 1 h, followed by 3–6 changes of TBST wash for 3 h at 4 °C. The resulting samples were heated at 95 °C for 10 min and applied to SDS-PAGE-based electrophoresis.

### Analysis of GA, HURP p725 by indirect immunofluorescence analysis

Cells seeded on coverslips were washed with PBS and fixed with periodate-lysine-paraformaldehyde containing 0.01 M periodate, 0.075 M lysine, 2% paraformaldehyde, 0.37 M phosphate buffer [30] at room temperature for 15 min. The fixed cells were incubated with permeabilization/blocking solution (75 mM NH4Cl, 20 mM Glycin pH8.0, 0.025% saponin, 0.2% BSA) at room temperature for 30 min. Sequentially, cells were incubated with primary antibodies at room temperature for 1 h, followed by 3 washes with TBST, and then incubated with DNA staining dye DAPI (4’,6-diamidino-2-phenylindole) (Sigma) and secondary antibody conjugated with Alexa Fluor 488 or Alexa Fluor 594 (Invitrogen) for 1 h at room temperature. After washing with TBST, the samples were mounted with Dako Mounting Medium (Agilent Technologies). The fluorescence images were analyzed using a fluorescence microscope (Leica DM2500) with Zyla sCMOS camera (Andor Technology). To analyze the structure of GA or HURP p725, 200 cells for each independent experiment were examined, and 3 independent experiments were performed. The GA structure was classified as the following criteria according to published papers [21, 49]: Ribbon, GA with continuous crescent shape; Compact, GA with ball-like appearance; Fragmentation, GA with discontinuous fragments scattering around the nucleus or cytoplasm. As to the measurement of the length of HURP p725 or GA, 20 cells were examined with the image analysis software MetaVue version 7.8.0.0 (Molecular Devices) for each independent experiment and 3 independent experiments were performed.

### In vitro methylation reaction

PRMT5-dependent methylation reaction was based on the methodology published elsewhere with modifications [49]. Briefly, the EGFP-PRMT5 and HURP were purified by immunoprecipitation adopting anti-GFP or HURP antibodies. The precipitates were rinsed two times with methylation buffer (50 mM Tri-HCl, pH 7.5, 1 mM EDTA and 1 mM EGTA), and then incubated with the methyl donor H^3^-S-adenosylmethionine in the presence of methylation buffer at 30 °C for 30 min, which was then stopped by adding SDS-PAGE sample buffer (Sigma).

### In vitro kinase reaction

The PRMT5 or HURP protein, obtained from immunoprecipitation, was incubated with recombinant His-Aurora-A in kinase reaction buffer (Tris HCl, pH 7.4, 10 mM MgCl2, 10 μM ATP, 2 mM EGTA, 1 mM DTT, 1 mM Na3VO4, 0.5 mM PMSF, 10% glycerol and with or without the presence of [γ-^32^P]-ATP at 30 °C for 30 min, which was then stopped by adding SDS-PAGE sample buffer. Dephosphorylation reaction was conducted employing His-λ phosphatase in buffer with 50 mM HEPES, pH7.5, 0.1 mM EDTA, 2 mM MnCl2 and 5 mM DTT at 30 °C for 30 min.

### Native-gel electrophoresis

Cells were lysed using the NativePAGE sample buffer (ThermoFisher Scientific) in the presence of 10% n-Dodecyl-β-D-maltoside (DDM) and 5% Digitonin. After centrifugation at 4 °C for 1 h, cell lysates were separated by gradient NativePAGE (4 to 16%). A buffer containing 0.5 M Tris (pH 9.2) and 0.5 M glycine was used for protein transfer. After transfer, the PVDF membrane was incubated in 20 ml of 7.5% acetic acid for 15 min at room temperature to fix the proteins. The membrane was subsequently rinsed with methanol and deionized water to remove the residual Coomassie blue G-250 dye. Western blots were then followed.

### Gel filtration

Cells were lysed with TNE buffer, containing 10 mM Tris–HCl (pH 7.8), 1% NP-40, 0.15 M NaCl, 1 mM EDTA, 1 mM phenylmethylsulfonyl fluoride (PMSF), and protease inhibitor cocktail. The cell lysates were then applied to a HiLoad 16/60 Superdex 75-pg column or a Superdex 75 10/300 GL column (GE Healthcare Life Science). The fractions were collected for further analyses.

### Statistical analysis

Student’s t-test was employed in all the experiments required for statistical analysis.

## Results

### PRMT5 methylates Aurora-A at R304 in the Ajuba-organized protein complex

**Fig. 1:**
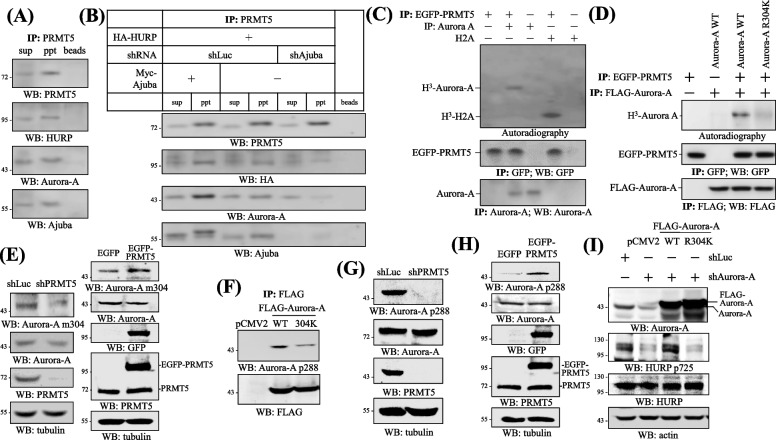
PRMT5 methylates Aurora-A in the Ajuba-organized protein complex. **A** Protein complex formation of Ajuba, PRMT5, Aurora-A and HURP. HeLa cells were applied to immune-coprecipitation assay adopting antibodies against PRMT5. Sup and ppt indicate supernatant and pellet. Western blots (WB) were followed to detect PRMT5, HURP, Aurora-A and Ajuba. **B** Manipulation of Ajuba expression causally determined formation of the Ajuba/PRMT5/Aurora-A/HURP complex. HeLa cells harboring HA-HURP were transfected with/or without Myc-Ajuba, or infected with viruses containing Luciferase shRNA (shLuc), Ajuba shRNA (shAjuba), and then subjected to immune-coprecipitation employing antibodies against PRMT5. Western blots were then followed to examine the formation of the protein complex. **C** PRMT5 methylated Aurora-A in vitro. To conduct an in vitro methylation reaction, immunoprecipitated Aurora-A or recombinant Histone 2A (H2A) was incubated with immunoprecipitated EGFP-PRMT5 in the presence of methylation reaction buffer containing H^3^ labelled methyl donor S-adenosylmethionine. The resulting samples were applied to autoradiography or Western blots to examine the methylation effect or immunoprecipitation results. **D** PRMT5 could not methylate Aurora-A R304K mutant. Immunoprecipitated FLAG-Aurora-A WT or R304K mutant was incubated with immunoprecipitated EGFP-PRMT5 for in vitro methylation reactions. Autoradiography or Western blot was performed to examine the methylation effect or immunoprecipitation results. **E** Knockdown or overexpression of PRMT5 reduced or elevated the level of Aurora-A m304. HeLa cells harboring shLuc, PRMT5 shRNA (shPRMT5) (left), or EGFP, EGFP-PRMT5 (right) were analyzed for the level of Aurora-A m304 and Aurora-A by Western blots. **F** The level of active Aurora-A was diminished on Aurora-A R304K. 293T cells transfected with pCMV2 empty vector, FLAG-Aurora-A WT or R304K were analyzed for the level of active Aurora-A, i.e. Aurora-A p288, on the immunoprecipitated FLAG-Aurora-A. **G** Knockdown of PRMT5 diminished the level of active Aurora-A. HeLa cells harboring shLuc or shPRMT5 were analyzed for the level of Aurora-A p288 and Aurora-A by Western blots. **H** Overexpression of PRMT5 elevated the level of active Aurora-A. 293 cells transfected with EGFP or EGFP-PRMT5 were analyzed for the expression of active Aurora-A and Aurora-A by Western blots. **I** Aurora-A R304K could not phosphorylate HURP. 293T cells were infected with shLuc or shAurora-A first, and the shAurora-A cells were further transfected with pCMV2 empty vector, FLAG-Aurora-A WT, R304K. These cells were then surveyed for the level of HURP p725, HURP and Aurora-A. Three independent experiments were performed for all the results listed above

To explore the potential contribution of protein modifications to GA assembly, we focused the following studies on two GA regulators with protein modifying activities, i.e., Aurora-A and PRMT5. We noticed that the scaffold protein Ajuba, localized to GA area according to the Human Protein ATLAS website (https://www.proteinatlas.org/ENSG00000129474-AJUBA/cell) on the basis of a study [43], is documented able to interact with Aurora-A [19] and PRMT5 [20], and our previous works show that Aurora-A phosphorylates HURP at S725 [48], and PRMT5 methylates HURP at R122 [11]. All these observations prompted us to speculate the potential formation of the protein complex containing Ajuba, PRMT5, Aurora-A and HURP. Indeed, Ajuba, Aurora-A and HURP were coprecipitated in immunoprecipitation assays employing antibodies against PRMT5 (Fig. 1A). Overexpression or knockdown of Ajuba enhanced or weakened interaction of the components in the protein complex (Fig. 1B), implying that Ajuba assembles the protein complex. To understand the molecular significance of the protein complex, we firstly found that PRMT5 methylated Aurora-A in vitro (Fig. 1C). We compared the protein sequence flanking the methylation site of 26 PRMT5’ substrates (Supplementary Figure 1), and a consensus sequence was deduced, i.e. NAl-X-NAl-R-NAl, where underlined R stands for the methylation site arginine, NAl represents nonpolar aliphatic amino acid, and X is any amino acid. Luckily, there is only one site that fits the consensus sequence in Aurora-A protein, namely ^301^I-E–G-R-M^305^. Subsequently, we performed site-directed mutagenesis and found that PRMT5 methylated Aurora-A R304K much less efficiently than methylated its wild type (WT) version (Fig. 1D). Furthermore, the antibodies against Aurora-A m304, i.e., the methylated form of Aurora-A at R304, were created, and knockdown (Fig. 1E, left) or overexpression (Fig. 1E, right) of PRMT5 decreased or increased the level of endogenous Aurora-A m304, collectively indicating that PRMT5 methylates Aurora-A at R304. Interestingly, compared to the Aurora-A WT, the Aurora-A R304K mutant had a lower level of Aurora-A p288 signal (Fig. 1F), the active form of Aurora-A [13], and knockdown (Fig. 1G) or overexpression (Fig. 1H) of PRMT5 reduced or increased the level of active Aurora-A without affecting the level of general Aurora-A. Furthermore, our previous studies show that Aurora-A phosphorylates HURP at serine residues including S725 [48]. Unlike the WT version of Aurora-A, the R304K mutant failed to restore the level of HURP p725 in Aurora-A knockdown cells (Fig. 1I). These lines of evidence together manifest themselves that PRMT5 methylates and in turn activates Aurora-A.

### Aurora-A phosphorylates PRMT5 at S103

**Fig. 2:**
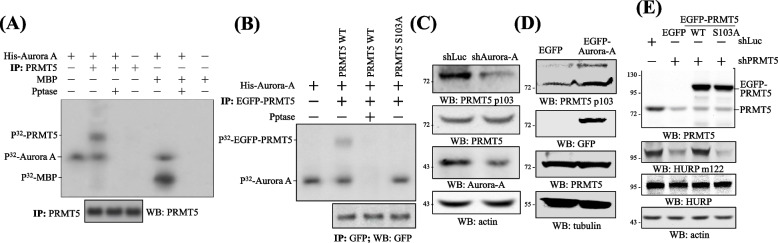
Aurora-A phosphorylates PRMT5 at S103. **A** PRMT5 served as an in vitro substrate of Aurora-A. To perform an in vitro kinase reaction, immunoprecipitated PRMT5 or recombinant MBP was incubated with recombinant His-Aurora-A in kinase reaction buffer containing P^32^-ATP. The resulting samples were applied to autoradiography or Western blots to examine the phosphorylation effect or the immunoprecipitation result. Pptase indicates alkaline phosphatase. Note, Aurora-A underwent autophosphorylation during the in vitro kinase reaction. **B** Aurora-A could not phosphorylate PRMT5 S103A. Immunoprecipitated EGFP-PRMT5 WT or S103A was incubated with recombinant His-Aurora-A for in vitro kinase reactions. Autoradiography or Western blots was conducted to examine the phosphorylation effect or the immunoprecipitation result. **C** Knockdown of Aurora-A decreased PRMT5 p103 in amount. HeLa cells harboring shLuc or shAurora-A were analyzed for the level of PRMT5 p103 and PRMT5 by Western blots. **D** Overexpression of Aurora-A increased the level of PRMT5 p103. 293 cells transfected with EGFP or EGFP-Aurora-A were surveyed for the level of PRMT5 p103 and PRMT5 by Western blots. **E** PRMT5 S103A could not methylate HURP. 293T cells were infected with shLuc or shPRMT5 first, and the shPRMT5 cells were further transfected with EGFP, EGFP-RPMT5 WT or S103A. These cells were then surveyed for the level of HURP m122 by Western blots. Three independent experiments were performed for all the results listed above

The interaction of Aurora-A and PRMT5 does not only facilitate PRMT5 to methylate Aurora-A, but also provides an opportunity for Aurora-A to phosphorylate PRMT5. As shown in Fig. 2A, Aurora-A could phosphorylate PRMT5 in vitro. To map the phosphorylation site, we firstly applied the PRMT5’s protein sequence to the public assessable phosphorylation prediction website, NetPhos 2.0 server (http://www.cbs.dtu.dk/services/NetPhos/), and S15, S16, S103, S273 and S446 were predicted as the most potential general phosphorylation sites with a score greater than 0.990. Secondly, the 5 sites were subjected to the website GPS 3.0-Kinase-specific Phosphorylation Site Prediction (http://gps.biocuckoo.org/), and PRMT5 S103 was predicted as the only potential phosphorylation site of Aurora-A. Indeed, the PRMT5 S103A could not be phosphorylated by Aurora-A (Fig. 2B). The antibodies against PRMT5 p103, i.e., the phosphorylated PRMT5 at S103, were then created, and knockdown (Fig. 2C) or overexpression (Fig. 2D) of PRMT5 diminished or elevated the level of endogenous PRMT5 p103. Our previous studies show that PRMT5 methylates HURP at R122 [11], and the current study revealed that unlike PRMT5 WT, the S103A mutant no longer restored the level of HURP m122 in PRMT5 knockdown cells (Fig. 2E). All these lines of evidence imply that Aurora-A is able to phosphorylate PRMT5 at S103, and which is crucial for PRMT5 to methylate HURP.

### PRMT5-induced HURP methylation is required for Aurora-A-catalyzed HURP phosphorylation

**Fig. 3:**
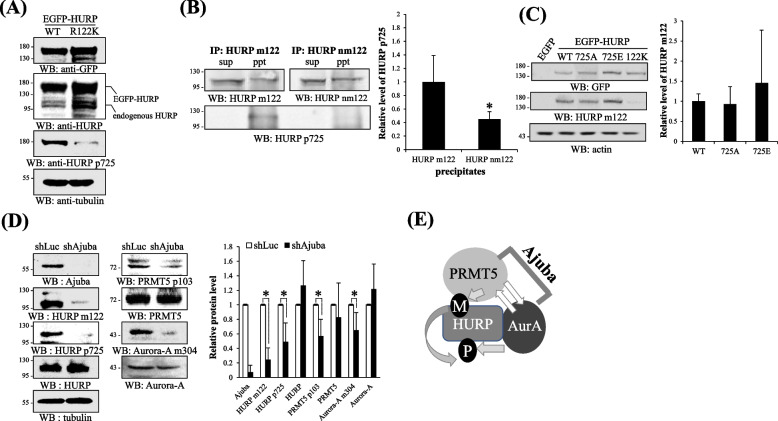
PRMT5-induced HURP methylation is required for Aurora-A-catalyzed HURP phosphorylation. **A** HURP p725 signal was barely detected on 122K mutant in cells. 293T cells transfected with EGFP-HURP WT or R122K were analyzed for the signal intensity of HURP p725 at the migration position on SDS-PAGE of EGFP-HURP. **B** HURP p725 signal was only detected on immunoprecipitated HURP m122. HURP m122 and HURP nm122, i.e. the HURP without methylation at R122, were immunoprecipitated in HeLa cells. Western blots were then followed to examine HURP p725 signal on the two precipitated HURPs. The HURP p725 signal was firstly normalized against HURP m122 or nm122; subsequently, the relative level of [nm122/m122]_p725_ was calculated and plotted. **C** No significant difference of HURP m122 signal on HURP WT, 725A and 725E. HeLa cells transfected with EGFP or various versions of EGFP-HURP were examined for the signal of HURP m122 at the EGFP-HURP position. The m122 signal was firstly normalized against EGFP-HURP; subsequently, the ratio of [m122/EGFP-HURP]_725A_ or [m122/EGFP-HURP]_725E_ to [m122/EGFP-HURP]_WT_ was calculated and plotted. **D** Knockdown of Ajuba decreased the level of HURP m122, HURP p725, PRMT5 p103 and Aurora-A m304. HeLa cells harboring shLuc or shAjuba were surveyed for the level of HURP m122, HURP p725, HURP, PRMT5 p103, PRMT5, Aurora-A m304, Aurora-A. The intensity of each protein was normalized against that of tubulin, and [shAjuba/shLuc]_Ajuba_, [shAjuba/shLuc]_HURP m122_, [shAjuba/shLuc]_HURP p725_ and [shAjuba/shLuc]_HURP_, [shAjuba/shLuc]_PRMT5 p103_, [shAjuba/shLuc]_PRMT5_, [shAjuba/shLuc]_Aurora-A m304_, and [shAjuba/shLuc]_Aurora-A_ were calculated and plotted. **E** The model for the component interaction in the Ajuba/PRMT5/Aurora-A/HURP complex. Ajuba keeps the two protein-modifying enzymes together, and PRMT5 and Aurora-A modify and activate each other. Subsequently, HURP is methylated by PRMT5 first, followed by Aurora-A (AurA)-mediated phosphorylation. Three independent experiments were performed for all the results listed above. * stands for statistical significance by Student’s t-test with *p* < 0.05

To investigate the potential mutual influence of the two modifications, it was found that HURP 122K could not undergo phosphorylation at S725 (Fig. 3A), and the HURP p725 signal was only detected on the HURP m122 antibodies-, rather than HURP nm122 antibodies-, based precipitates in immunoprecipitation assays (Fig. 3B), where HURP nm122 stands for the HURP not being methylated at R122. The HURP m122 antibodies and nm122 antibodies had no or very weak cross-reaction (Supplementary Figure 2). On the contrary, the level of HURP m122 signal on HURP 725A was similar to that of HURP WT (Fig. 3C), together implying that the modification of HURP p725 requires the presence of HURP m122 but not the vice versa. Furthermore, depletion of Ajuba largely reduced the level of HURP m122, HURP p725, PRMT5 p103 and Aurora-A m304 without affecting the general HURP, PRMT5 and Aurora-A (Fig. 3D), indicating that the modifications of HURP p725, PRMT5 p103 and Aurora-A m304 rely on the formation of the complex Ajuba/PRMT5/Aurora-A. All these collected data indicate that, Ajuba keeps PRMT5 and Aurora-A together, allowing them to activate each other, and sequentially HURP p725 is produced (Fig. 3E).

### HURP p725 is required for the formation of crescent Golgi ribbon

**Fig. 4:**
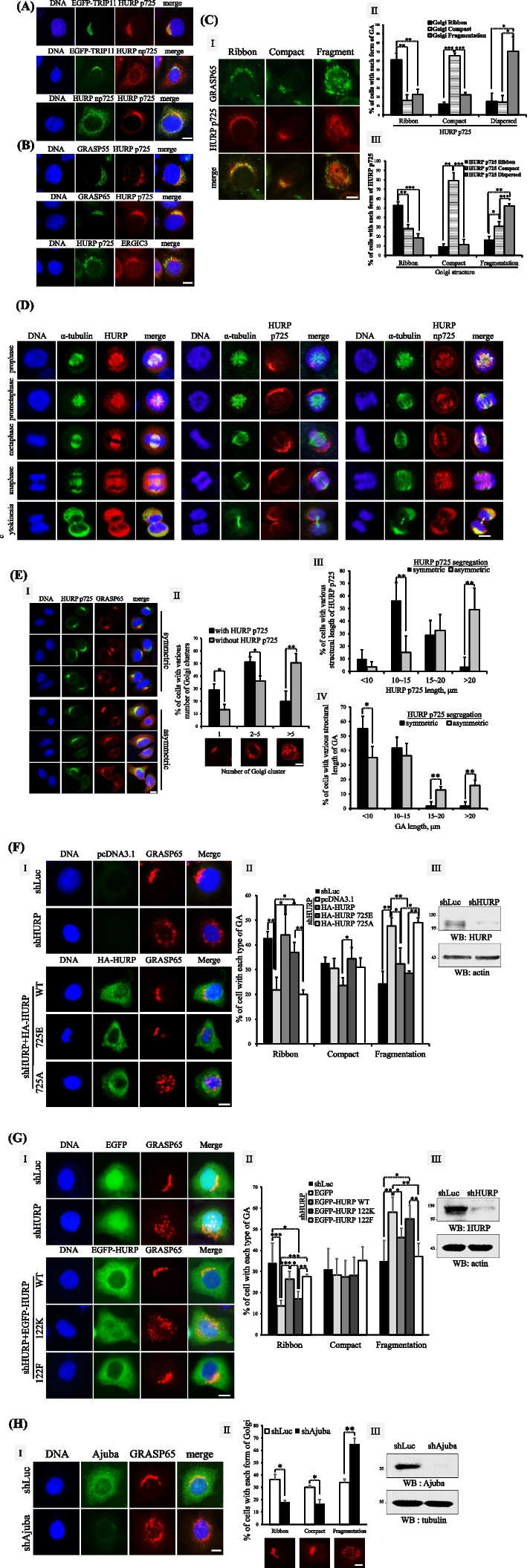
HURP p725 is required for the formation of Golgi ribbon. **A** Differential localization of HURP p725 and np725. HeLa cells were examined for the subcellular localization of HURP p725 and np725 by immunofluorescence. EGFP-TRIP11 was used to label GA. Three independent experiments were performed. **B** Colocalization of HURP p725 with cis-Golgi marker. Immunofluorescence was conducted in HeLa cells to visualize HURP p725, trans-Golgi (GRASP55), cis-Golgi (GRASP65) and the ER-Golgi intermediate compartment (ERGIC3). Three independent experiments were performed. **C** HURP was localized to GA area and displayed morphological correlation with GA. Immunofluorescence was conducted in HeLa cells to visualize HURP p725 and GA (GRASP65) (I). The cells with HURP p725 in crescent ribbon-like bundle (ribbon), compact or fragment were selectively examined, and % of those selected cells having GA in crescent ribbon, compact or fragment form was counted and plotted (II). By contrast, the cells with GA in crescent ribbon (ribbon), compact or fragment were selectively examined, and % of those selected cells having HURP p725 in crescent ribbon-like bundle (ribbon), compact or fragment form was counted and plotted (III). To analyze the structure of GA or HURP p725, 200 cells for each independent experiment were examined, and 3 independent experiments were performed. **D** HURP p725 displayed extended fiber-like distribution and np725 was localized to spindle in mitotic cells. HeLa cells were applied to immunofluorescence to visualize the distribution of HURP, HURP p725, np725 and spindle. Mitotic cells were selectively examined. Three independent experiments were performed. **E** The cells losing HURP p725 or containing longer HURP p725 fiber had more fragmented or elongated GA. The HeLa cell doublets undergoing cytokinesis were selectively examined, including cells with symmetric or asymmetric segregation of HURP p725. HURP p725 and GA (GRASP65) were detected by immunofluorescence (I). The % of cells with or without HURP p725 containing GA fragments distributed in 1, 2–5 or greater than 5 clusters were counted and plotted (II). The length of HURP p725 (III) or GA (IV) in cell doublets with symmetric or asymmetric segregation of HURP p725 was measured. The length of HURP p725 or GA in the cell doublet containing symmetric HURP p725 was the average of the two connected cells. Length of GA or HURP p725 was measured only in the cells with HURP p725 when HURP p725 was asymmetrically segregated. To analyze the structure of GA or HURP p725, 200 cells for each independent experiment were examined, and 3 independent experiments were performed. As to the measurement of the length of HURP p725 or GA, 20 cells were examined with the image analysis software MetaVue version 7.8.0.0 (Molecular Devices) for each independent experiment and 3 independent experiments were performed. **F** Knockdown of HURP fragmented GA, and that was not rescued by HURP 725A. HeLa cells harboring shLuc or shHURP plus each one of pcDNA3.1 empty vector, HA-HURP WT, 725E or 725A were analyzed for the GA structure by immunofluorescence (I, II), or knockdown efficiency by Western blot (III). The % of cells with various forms of GA was counted and plotted. To analyze the structure of GA stained by GRAST65 antibodies, 200 cells either with shLuc, or shHURP plus HA-HURP WT, 725E or 725A, for each independent experiment were examined, and 3 independent experiments were performed. **G** HURP 122K lost the Golgi ribbon constructing activity. HeLa cells harboring shLuc, shHURP plus EGFP, or shHURP plus each one of EGFP-HURP versions were analyzed for the GA structure by immunofluorescence (I, II), or knockdown efficiency by Western blot (III). The % of cells with various forms of GA was counted and plotted. To analyze the structure of GA stained by GRAST65 antibodies, 200 cells either with shLuc, or shHURP plus HA-HURP WT, 122K or 122F, for each independent experiment were examined, and 3 independent experiments were performed. **H** Knockdown of Ajuba fragmented GA. HeLa cells harboring shLuc or shAjuba were analyzed for the GA structure by immunofluorescence (I, II), or knockdown efficiency by Western blot (III). To analyze the structure of GA stained by GRAST65 antibodies, 200 cells either with shLuc or shAjuba for each independent experiment were examined, and 3 independent experiments were performed. The % of cells with various forms of GA was counted and plotted. *, **, and *** stand for statistical significance by Student’s t-test with *p* < 0.05, 0.01 and 0.001 respectively. Scale bar: 10 μm

To explore the cellular functions of HURP p725, we detected the subcellular localization of HURP p725 and np725, the HURP without phosphorylation at S725. There was no cross-reaction between HURP p725 antibodies and np725 antibodies (Supplementary Figure 3). Unlike the cytoplasmic distribution of np725, HURP p725 was localized to GA region in interphase cells (Fig. 4A), and had spatial distribution closer to cis-Golgi (Fig. 4B). Besides, the distribution pattern of HURP p725 resembled GA structure (Fig. 4C). For example, when the cells with HURP p725 in crescent-like ribbon (simply designated as ribbon hereafter) were selectively examined, 60% of these cells had their GA in crescent ribbon shape (ribbon) (Fig. 4C-I, II). Alternatively, more than 50% of cells with Golgi in ribbon form had HURP p725 in ribbon-like shape (Fig. 4C-I, III). To unravel the potential contribution of HURP p725 to GA formation, we found that HURP p725, rather than np725 which localized to the spindle, distributed as a long fiber-like pattern in early mitosis, and two shorter HURP p725 fiber-like segments were detected during cytokinesis (Fig. 4D), where it is known that GA begins to reform from tiny Golgi membranes. Interestingly, HURP p725 did not always symmetrically segregate into two daughter cells in a cell doublet during cytokinesis in HeLa cells. HURP p725 was sometimes detected in only one cell of a cell doublet. We seeded cells with low density, so that cells did not contact each other. Subsequently, we examined the post-mitotic cell doublets, which contained two smaller connected cells, with asymmetrically segregated HURP p725, i.e., HURP p725 was absent in one daughter cell of a cell doublet, and found that the cell losing HURP p725 had GA membranes scattering around the cytoplasm, while the other cell possessing HURP p725 had GA membranes with a higher compacting tendency (Fig. 4E-I, II). The length of HURP p725 segment in the cell asymmetrically obtaining HURP p725 of a cell doublet was much longer than that in the cell doublet symmetrically or equally gaining HURP p725 (Fig. 4E-I, III); subsequently, the cells with longer HURP p725 also had a longer GA structure (Fig. 4E-I, IV). These data reveal a structural correlation of HURP p725 and GA status. Further causal study adopting site-directed mutagenesis showed that GA was fragmented in HURP knockdown cells, and HURP WT and the phosphorylation mimicking mutant 725E could restore the Golgi ribbon. By contrast, the phosphorylation deficiency mutant 725A could not rescue Golgi ribbon in HURP knockdown cells (Fig. 4F). Besides, the HURP methylation deficiency mutant 122K lost the Golgi ribbon constructing activity (Fig. 4G) and knockdown of Ajuba disorganized GA (Fig. 4H), in line with the finding that HURP m122 is required for the production of HURP p725 in the Ajuba-organized protein complex.

### HURP p725 regulates the localization and protein stability of TRIP11

**Fig. 5:**
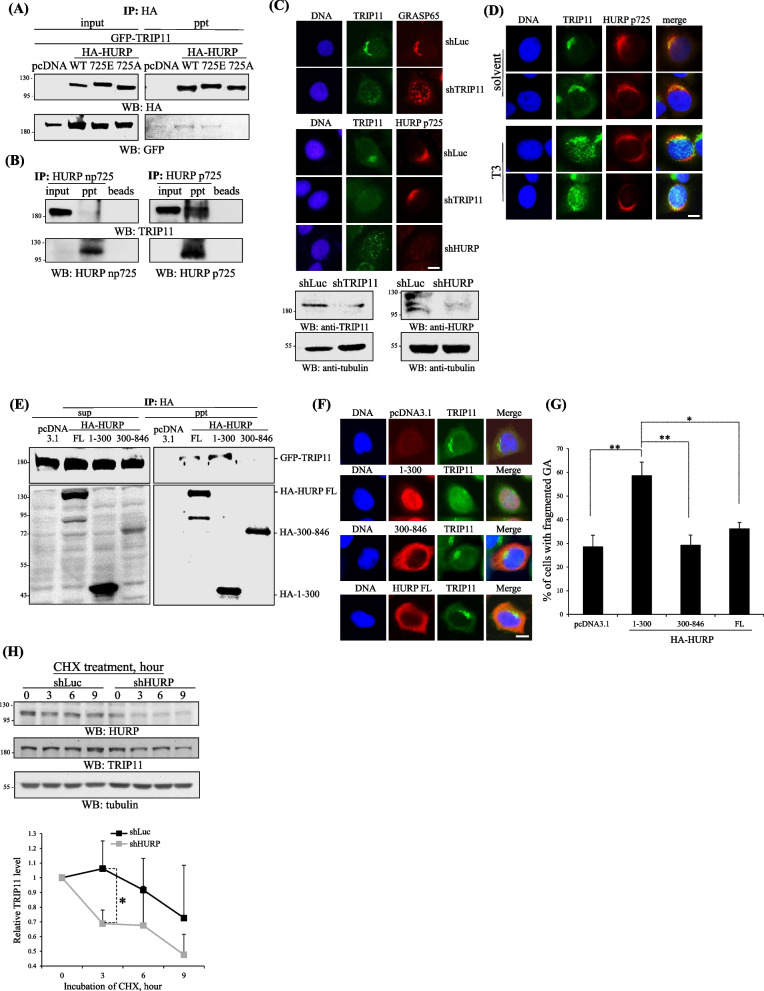
HURP p725 regulates the localization and protein stability of TRIP11. **A** Interaction of GFP-TRIP11 with HA-HURP. 293T cells transfected with GFP-TRIP11 and various versions of HA-HURP were analyzed for the interaction of TRIP11 and HURP by performing immune-coprecipitation. pcDNA is the empty vector pcDNA3.1. Three independent experiments were performed. **B** Interaction of HURP p725 and TRIP11. Immuno-coprecipitation was conducted in HeLa cells to examine the interaction of endogenous TRIP11 and HURP p725 or np725. Three independent experiments were performed. **C** HURP was required for maintaining the GA localization of TRIP11 and not the vice versa. HeLa cells with shHURP or TRIP11 shRNA (shTRIP11) were analyzed for the knockdown efficiency (lower) or subcellular distribution of HURP p725 or TRIP11 (upper). Three independent experiments were performed. **D** Translocation of TRIP11 to nucleus did not affect subcellular localization of HURP p725. HeLa cells treated with solvent or T3 (2 μM, 2 h) were surveyed for the subcellular distribution of HURP p725 or TRIP11. Three independent experiments were performed. **E** HURP employed its N-terminal to interact with TRIP11. 293 cells, transfected with GFP-TRIP11 and each one of empty vector pCDNA3.1, HA tagged HURP full length (FL), 1–300 or 300–846, were analyzed for the interaction with TRIP11 by conducting immune-coprecipitation using antibodies against HA. Three independent experiments were performed. **F** The nuclear localization of HURP 1–300 attracted TRIP11 into nucleus. HeLa cells, harboring HA-HURP FL, 1–300, 300–846, were examined for subcellular localization of TRIP11 by performing immunofluorescence. Three independent experiments were performed. **G** The HURP deletion mutant 1–300 attracting TRIP11 into the nucleus disassembled GA. HeLa cells harboring HURP full length (FL), 1–300 or 300–846 were examined for GA structure by conducting immunofluorescence adopting antibodies against GRASP55. To analyze the structure of GA stained by GRAST65 antibodies, 200 cells either with pcDNS3.1, HA-HURP 1–300, 300–846 or FL, for each independent experiment were examined, and 3 independent experiments were performed. **H** Knockdown of HURP destabilized TRIP11. HeLa cells with shLuc or shHURP were treated with cycloheximide (CHX) to inhibit protein de novo synthesis for 0–9 h. Western blots were then followed to determine the protein stability of TRIP11. Three independent experiments were conducted. *, **, and *** stand for statistical significance by Student’s t-test with *p* < 0.05, 0.01 and 0.001 respectively. Scale bar: 10 μm

To understand how HURP p725 dictates the formation of Golgi ribbon, we found a cis-Golgi protein TRIP11 [22], selectively interacted with HURP WT, 725E or endogenous HURP p725, while did not bind 725A or np725 (Fig. 5A and B). Knockdown of TRIP11 disassembled GA and did not disturb the distribution of HURP p725 (Fig. 5C), not only implying that TRIP11 does not guide the subcellular distribution of HURP p725, but also revealing that localization of HURP p725 to GA area does not rely on the presence or structural integrity of Golgi ribbon. In support of that notion, the thyroid hormone triiodothyronine (T3)-induced nuclear targeting of TRIP11 did not move HURP p725 into the nucleus (Fig. 5D). By contrast, silence of HURP mislocalized TRIP11 (Fig. 5C). Moreover, overexpression of the HURP deletion mutant 1–300, which retained the interaction domain for TRIP11 (Fig. 5E) and localized to the nucleus (Fig. 5F), forced the GAF, TRIP11, away from GA and targeting to the nucleus (Fig. 5F), which in turn fragmentized GA (Fig. 5G). Further study revealed that TRIP11 protein was unstable in HURP depletion cells (Fig. 5H), collectively suggesting that HURP p725 interacts with, stabilizes and dictates localization of TRIP11.

### HURP p725 binds and stabilizes more cis-GAFs

**Fig. 6:**
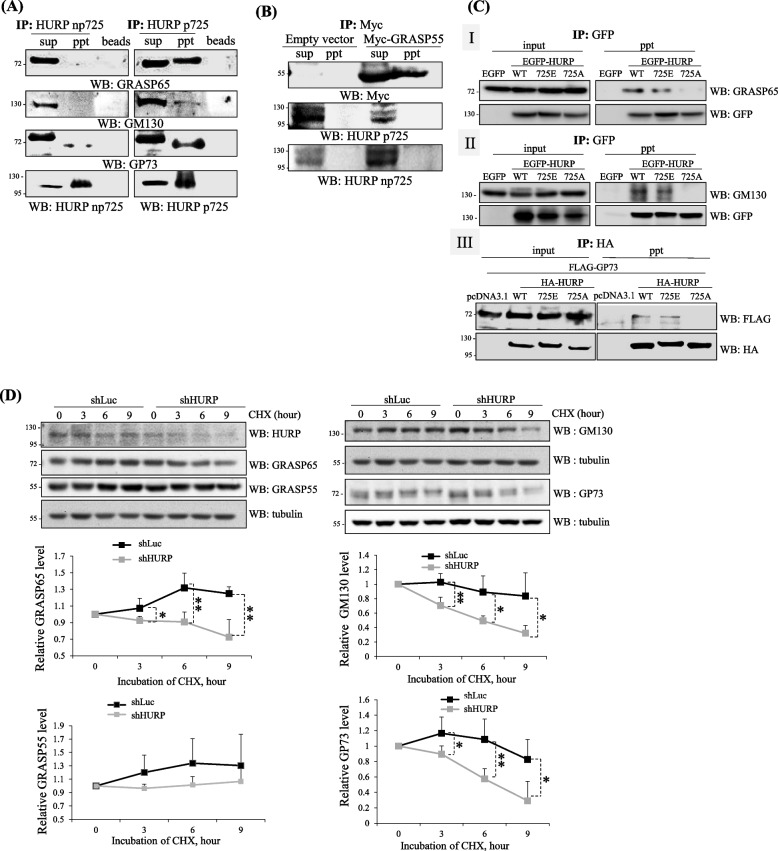
HURP p725 binds and stabilizes more cis-GAFs. **A** HURP p725 interacted with three cis-Golgi proteins. HeLa cells were applied to HURP p725 antibodies or np725 antibodies-based immuno-coprecipitation, and Western blots adopting antibodies against GRASP65, GM130, or GP73 were performed. Three independent experiments were performed. **B** HURP p725 did not bind GRASP55. Immuno-coprecipitation was performed in HeLa cells harboring empty vector or Myc-GRASP55 to detect the interaction of GRASP55 and HURP p725 or np725. Three independent experiments were performed. **C** Interaction of EGFP-HURP with the three cis-GAFs. 293 cells harboring EGFP, EGFP-HURP WT, 725E or 725A were analyzed for the interaction of EGFP-HURP with GRASP65 (I), GM130 (II) and GP73 (III). Three independent experiments were performed. **D** Knockdown of HURP destabilized GRASP65, GM130 and GP73, while did not affect GRASP55. Protein stability of the four Golgi proteins was surveyed in shLuc and shHURP cells treated with cycloheximide. Three independent experiments were conducted to obtain the relative protein level of each protein. *, **, and *** stand for statistical significance by Student’s t-test with *p* < 0.05, 0.01 and 0.001 respectively

In addition to TRIP11, HURP p725 also interacted with several other cis-GAFs, such as GRASP65 [44], GM130 [33] and GP73 [3] (Fig. 6A), and did not bind to the trans-GAF such as GRASP55 [38] (Fig. 6B). Consistently, GRASP65, GM130 and G73 bound to EGFP-HURP WT and 725E, while did not interact with 725A (Fig. 6C). Similarly, those cis-GAFs, rather than trans-GAF GRASP55, had reduced protein stability in HURP knockdown cells (Fig. 6D). Taking all the pieces of evidence together, HURP p725 assembles GA by binding and stabilizing those cis-GAFs.

### ARF1/Golgin-160 controls the subcellular localization of HURP p725

**Fig. 7:**
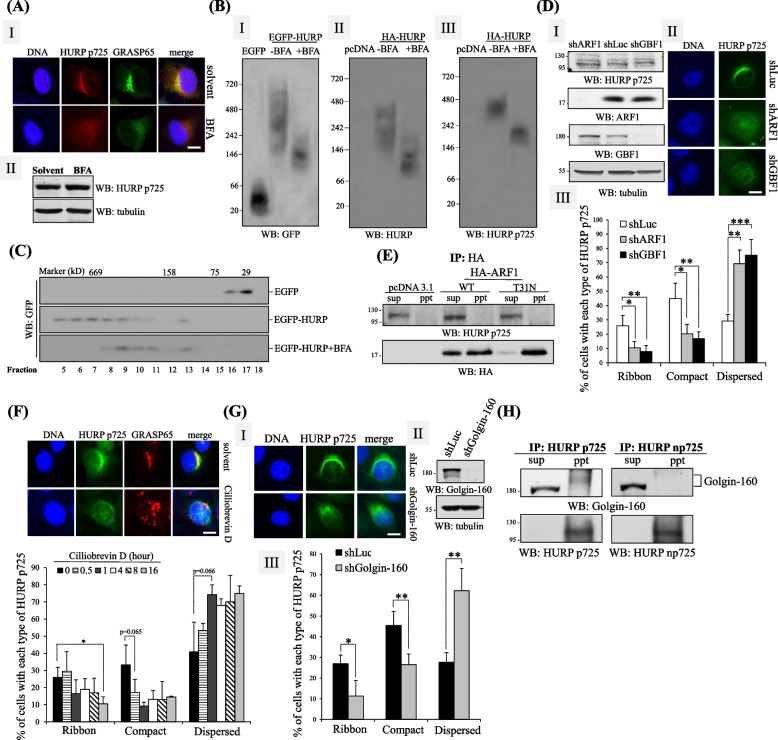
ARF1/Golgin-160 controls subcellular distribution of HURP p725. **A** BFA disorganized HURP p725 and GA. HeLa cells treated with or without 5 μM BFA for 1 h were visualized for GA morphology (BRAST65) or subcellular distribution (I) and protein level (II) of HURP p725 by immunofluorescence and Western blot. Three independent experiments were performed. **B** Native gel analysis of HURP and HURP p725. HeLa cells transfected EGFP, EGFP-HURP (I), or pcDNA3.1 empty vector, HA-HURP (II, III) were treated with or without BFA. The resulting samples were applied to native gel-based electrophoresis followed by performing Western blots to detect GFP (I), HURP (II) or HURP p725 (III). Three independent experiments were performed. **C** Gel filtration assay for HURP. HeLa cells transfected with EGFP or EGFP-HURP were treated with or without BFA. Subsequently, the cell extracts of fraction 5–18 from gel filtration were collected and Western blots adopting GFP antibodies were performed. Three independent experiments were performed. **D** Knockdown of ARF1 or GBF1 disorganized HURP p725. HeLa cells harboring shLuc, ARF1 shRNA (shARF1) or GBF1 shRNA (shGBF1) were examined for the knockdown efficiency of the two proteins by Western blots (I) or the subcellular distribution of HURP p725 by immunofluorescence (II). The % of cells with each type of HURP p725 distribution was counted and plotted (III). To analyze the distribution of HURP p725, 200 cells either with shLuc, shARF1 or GBF1 for each independent experiment were examined, and 3 independent experiments were performed. **E** ARF1 did not interact with HURP p725. Immuno-coprecipitation was performed to detect the interaction of HA-tagged ARF1 WT or inactive mutant T31N with HURP p725. Three independent experiments were performed. **F** Dynein inhibitor disorganized HURP p725. HeLa cells treated with 50 μM dynein inhibitor Cilliobrevin D for 0–16 h were applied to immunofluorescence to detect subcellular localization of HURP p725. The % of cells with each kind of HURP p725 distribution was counted and plotted. To analyze the distribution pattern of HURP p725, 200 cells treated with Cilliobrevin for different times for each independent experiment were examined, and 3 independent experiments were performed. **G** Knockdown of Golgin-160 dispersed HURP p725. HeLa cells harboring shLuc or Golgi-160 shRNA (shGolgin-160) were examined for HURP p725 distribution (I) or knockdown down efficiency (II). The % of cells with each kind of HURP p725 distribution was counted and plotted (III). To analyze the distribution pattern of HURP p725, 200 cells either with shLuc or shGolgin-160 for each independent experiment were examined, and 3 independent experiments were performed. **H** Golgin-160 interacted with HURP p725. Immuno-coprecipitation employing antibodies against HURP p725 or np725 was conducted in HeLa cells to examine the interaction of HURP and Golgin-160. Three independent experiments were performed. *, **, and *** stand for statistical significance by Student’s t-test with *p* < 0.05, 0.01 and 0.001 respectively. Scale bar: 10 μm

To further investigate the mechanisms by which HURP p725 is organized as a crescent-like structure and integrate the HURP p725-mediated regulation of Golgi ribbon formation to the known GA regulatory networks, we began by addressing how HURP p725 is localized to the vicinity of GA. It was found firstly that the Golgi disrupting agent BFA [37] dispersed the distribution, and did not decrease the protein level, of HURP p725 (Fig. 7A). The dispersal of HURP p725 localization was not caused by the structural disruption of GA, because knockdown of TRIP11-induced Golgi disassembly did not interfere with the localization of HURP p725 (Fig. 5C). Moreover, the HURP molecules, no matter tagged by EGFP or HA, migrated on native gels with various sizes ranging from 146 kD to 720 kD (Fig. 7B). Treatment of BFA dramatically reduced the heterogeneity of HURP and shifted the electrophoretic position of HURP to the molecular weight of 66 ~ 146 kD, roughly equivalent to the molecular weight of a single HURP molecule (Fig. 7B, left and middle). Interestingly, HURP p725 was found migrating at the highest position among the whole HURP species in the native gel electrophoresis (Fig. 7B, right), and its mobility was also enhanced by BFA, hinting the BFA-sensitive HURP p725 is organized as a high-order structure in cells. Similarly, gel filtration studies detected the majority of EGFP-HURP in the fractions of cell extracts with molecular weight near or higher than 669 kD; nevertheless, HURP was observed in the fractions with molecular weight lower than 669 kD when cells were exposed to BFA (Fig. 7C). To further uncover how BFA disturbs HURP p725, it was learned that BFA directly binds and inactivates ARF1 and its GTP/GDP exchange factor GBF1 [36]. Silence of ARF1 or GBF1 turned the ribbon-like bundle of HURP p725 into dispersed form (Fig. 7D), however, ARF1 did not interact with HURP p725 (Fig. 7E), implying that ARF1 controls HURP p725 indirectly. Given that ARF1 gathers small Golgi membranes via recruiting the Dynein cargo adaptor Golgin-160, chemical inhibition of cytoplasmic Dynein (Fig. 7F) or silence of Golgin-160 (Fig. 7G) dispersed HURP p725, and Golgin-160 interacted with HURP p725, and did not bind to np725 (Fig. 7H). All these findings collectively indicate that the Golgin-160/Dynein complex regulates subcellular localization of HURP via binding and transporting HURP under the guidance of ARF1.

## Discussion

### The Ajuba/PRMT5/Aurora-A complex integrates the signals of protein methylation and phosphorylation to HURP

Ajuba is originally identified as an Aurora-A activator [19] and the activation mechanisms have been reported later on [4]. On the other hand, the activation mechanisms of PRMT5 remain elusive [24]. We here identify a distinct mechanism for how Ajuba activates Aurora-A and how PRMT5 is activated, where Ajuba assembles the PRMT5 and Aurora-A complex, and reciprocal modifications of the two enzymes trigger the activation of them. Hence, Ajuba integrates the signaling from protein phosphorylation and methylation via scaffolding Aurora-A and PRMT5, and the signals are relayed to their common substrate HURP. It is likely that methylation at N-terminal (R122) and phosphorylation at C-terminal (S725) of HURP which contains 846 amino acids, could induce dramatic conformational changes entirely by increasing the hydrophobicity and hydrophilicity of the two ends of HURP simultaneously, thereby exposing its GAF-interacting domain and in turn promoting the assembly of crescent Golgi ribbon by recruiting and stabilizing GAFs.

### HURP p725 assembles Golgi ribbon from GA cis-side

The GA structure is maintained in a dynamic equilibrium between input and output of membranes from and to other organelles, including the endoplasmic reticulum (ER), the endosome–lysosome system, and the plasma membrane [26]. The COPI vesicles from ER are captured and tethered to the cis-cisternae by the tertiary Giantin-p115-GM130 tethering complex prior to membrane fusion during GA assembly [32]. Moreover, the cis-GAF Golgin-160 centripetally transports Golgi vesicles also to the cis-Golgi [46]. All these observations indicate that GA structurally grows at its cis-side. In support of this notion, our analyses show that the HURP p725 localizes to the cis-Golgi and binds cis-GAFs rather than trans-GAFs such as GRASP55. Once the GA membrane-associated GAFs are caught by HURP p725, they become stabilized and execute their functions on where HURP p725 resides. The HURP p725-regulated cis-GAFs such as GM130, GRASP65 and TRIP11, which function in promoting fusion between GA membrane and vesicles and lateral fusion of cisternae [34], stacking flattened cisterna and linking of stacks [5, 34], and fusion between curved and flattened membranes and lateral linking between stacks [14, 35], respectively. Hence, all these HURP p725 interacted GAFs function in regulating the Golgi forming process from early to late stage, thereby ensuring GA assembly from its cis-side on the surface of HURP p725.

## Conclusion

The Ajuba/PRMT5/Aurora-A complex integrates the signals of protein methylation and phosphorylation to HURP, and the HURP p725 organizes GA by stabilizing and recruiting GAFs to its crescent-like structure, therefore shaping GA as a crescent ribbon. Therefore, the HURP p725 fiber serves a template to construct GA according to its shape.

## Supplementary Information

**Additional file 1: Supplementary Figure 1. **Deduction of PRMT5-dependent methylation determinant sequence. **Supplementary Figure 2.** The HURP m122 antibodies and nm122 antibodies almost did not cross react. **Supplementary Figure 3.** Antibody specificity of HURP p725 and np725.

## Data Availability

The data used to support the findings of this study are included within the article.

## Abbreviations

GA: Golgi apparatus
GAFs: Golgi assembly factors
WT: Wild type
ribbon: Crescent ribbon shape
ER: Endoplasmic reticulum
Brefeldin A: BFA
