# Supplementary material for: The crescent-like Golgi ribbon is shaped by the Ajuba/PRMT5/Aurora-A complex-modified HURP
Source: Cell Commun Signal. 2023 Jun 27;21:156. doi: 10.1186/s12964-023-01167-4 (PMC10294536; doi:10.1186/s12964-023-01167-4)
Supplement: Supplementary file 2 — Additional file 1: Supplementary Figure 1. Deduction of PRMT5-dependent methylation determinant sequence. Supplementary Figure 2. The HURP m122 antibodies and nm122 antibodies almost did not cross react. Supplementary Figure 3. Antibody specificity of HURP p725 and np725. [file 12964_2023_1167_MOESM1_ESM.pdf]

Supplementary Figure 1

PRMT5's substrates	a.a. site -3	-2	-1	0	1	Ref
SPT5 R698	S	R	G	R	G	a
p53 R333	L	Q	I	R	G	b
p53 R335	I	R	G	R	E	b
p53 R337	G	R	E	R	F	b
CBP-1 R234	P	M	G	R	G	c
ASH2L R296	G	K	G	R	G	d
PDCD4 R110	G	K	G	R	G	e
HoxA R140	S	A	R	R	G	f
NF-κB R30	P	K	Q	R	G	g
E2F1 R109	G	P	A	R	G	h
E2F1 R111	A	R	G	R	G	h
E2F1 R113	G	R	G	R	H	h
GM130 R6	E	E	T	R	Q	i
GM130 R18	K	K	L	R	E	i
GM130 R23	Y	Q	Q	R	N	i
RPS10 R158	G	F	G	R	G	j
RPS10 R160	G	R	G	R	G	j
FCP1 R913	A	G	G	R	G	k
FCP1 R916	R	G	P	R	G	k
FEN1 R19	S	A	I	R	E	l
FEN1 R100	L	A	K	R	S	l
FEN1 R104	S	E	R	R	A	l
FEN1 R192	V	L	M	R	H	l
RAD9 R172	G	I	G	R	G	m
RAD9 R174	G	R	G	R	R	m
RAD9 R175	R	G	R	R	V	m
**Consensus**	**NAl**	**X**	**NAl**	**R**	**NAl**	
Frequency	58%		65%		62%	

Nonpolar aliphatic	GAVLMI
Aromatic	FYW
Polar uncharged	STCPNQ
Positive charged	KRH
Negative charged	DE

Ref	
a	Kwak YT, Guo J, Prajapati S, Park KJ, Surabhi RM, Miller B, Gehrig P, Gaynor RB. Methylation of SPT5 regulates its interaction with RNA polymerase II and transcriptional elongation properties.
b	Jansson M, Durant ST, Cho EC, Sheahan S, Edelmann M, Kessler B, La Thangue NB. Arginine methylation regulates the p53 response.
b	Jansson M, Durant ST, Cho EC, Sheahan S, Edelmann M, Kessler B, La Thangue NB. Arginine methylation regulates the p53 response.
b	Jansson M, Durant ST, Cho EC, Sheahan S, Edelmann M, Kessler B, La Thangue NB. Arginine methylation regulates the p53 response.
c	Yang M, Sun J, Sun X, Shen Q, Gao Z, Yang C. Caenorhabditis elegans protein arginine methyltransferase PRMT-5 negatively regulates DNA damage-induced apoptosis. PLoS Genet. 2009 Jun;5(6):e1000514.
d	Butler JS, Zurita-Lopez CI, Clarke SG, Bedford MT, Dent SY. Protein-arginine methyltransferase 1 (PRMT1) methylates Ash2L, a shared component of mammalian histone H3K4 methyltransferase complexes. J Biol Chem. 2011 Apr 8;286(14):12234-44.
e	Powers MA, Fay MM, Factor RE, Welm AL, Ullman KS. Protein arginine methyltransferase 5 accelerates tumor growth by arginine methylation of the tumor suppressor programmed cell death 4. Cancer Res. 2011 Aug 15;71(16):5579-87.
f	Dhar SS, Lee SH, Kan PY, Voigt P, Ma L, Shi X, Reinberg D, Lee MG. Trans-tail regulation of MLL4-catalyzed H3K4 methylation by H4R3 symmetric dimethylation is mediated by a tandem PHD of MLL4. Genes Dev. 2012 Dec 15;26(24):2749-62.
g	Wei H, Wang B, Miyagi M, She Y, Gopalan B, Huang DB, Ghosh G, Stark GR, Lu T. PRMT5 dimethylates R30 of the p65 subunit to activate NF-κB. Proc Natl Acad Sci U S A. 2013 Aug 13;110(33):13516-21.
h	Cho EC, Zheng S, Munro S, Liu G, Carr SM, Moehlenbrink J, Lu YC, Stimson L, Khan O, Konietzny R, McGouran J, Coutts AS, Kessler B, Kerr DJ, Thangue NB. Arginine methylation controls growth regulation by E2F-1. EMBO J. 2012 Apr 4;31(7):1785-97.
h	Cho EC, Zheng S, Munro S, Liu G, Carr SM, Moehlenbrink J, Lu YC, Stimson L, Khan O, Konietzny R, McGouran J, Coutts AS, Kessler B, Kerr DJ, Thangue NB. Arginine methylation controls growth regulation by E2F-1. EMBO J. 2012 Apr 4;31(7):1785-97.
h	Cho EC, Zheng S, Munro S, Liu G, Carr SM, Moehlenbrink J, Lu YC, Stimson L, Khan O, Konietzny R, McGouran J, Coutts AS, Kessler B, Kerr DJ, Thangue NB. Arginine methylation controls growth regulation by E2F-1. EMBO J. 2012 Apr 4;31(7):1785-97.
i	Zhou Z, Sun X, Zou Z, Sun L, Zhang T, Guo S, Wen Y, Liu L, Wang Y, Qin J, Li L, Gong W, Bao S. PRMT5 regulates Golgi apparatus structure through methylation of the golgin GM130. Cell Res. 2010 Sep;20(9):1023-33.
i	Zhou Z, Sun X, Zou Z, Sun L, Zhang T, Guo S, Wen Y, Liu L, Wang Y, Qin J, Li L, Gong W, Bao S. PRMT5 regulates Golgi apparatus structure through methylation of the golgin GM130. Cell Res. 2010 Sep;20(9):1023-33.
i	Zhou Z, Sun X, Zou Z, Sun L, Zhang T, Guo S, Wen Y, Liu L, Wang Y, Qin J, Li L, Gong W, Bao S. PRMT5 regulates Golgi apparatus structure through methylation of the golgin GM130. Cell Res. 2010 Sep;20(9):1023-33.
j	Ren J, Wang Y, Liang Y, Zhang Y, Bao S, Xu Z. Methylation of ribosomal protein S10 by protein-arginine methyltransferase 5 regulates ribosome biogenesis. J Biol Chem. 2010 Apr 23;285(17):12695-705.
j	Ren J, Wang Y, Liang Y, Zhang Y, Bao S, Xu Z. Methylation of ribosomal protein S10 by protein-arginine methyltransferase 5 regulates ribosome biogenesis. J Biol Chem. 2010 Apr 23;285(17):12695-705.
k	Amente S, Napolitano G, Licciardo P, Monti M, Pucci P, Lania L, Majello B. Identification of proteins interacting with the RNAPII FCP1 phosphatase: FCP1 forms a complex with arginine methyltransferase PRMT5 and it is a substrate for PRMT5-mediated methylation. FEBS Lett. 2005 Jan 31;579(3):683-9.
k	Amente S, Napolitano G, Licciardo P, Monti M, Pucci P, Lania L, Majello B. Identification of proteins interacting with the RNAPII FCP1 phosphatase: FCP1 forms a complex with arginine methyltransferase PRMT5 and it is a substrate for PRMT5-mediated methylation. FEBS Lett. 2005 Jan 31;579(3):683-9.
l	Guo Z, Zheng L, Xu H, Dai H, Zhou M, Pascua MR, Chen QM, Shen B. Methylation of FEN1 suppresses nearby phosphorylation and facilitates PCNA binding. Nat Chem Biol. 2010 Oct;6(10):766-73.
l	Guo Z, Zheng L, Xu H, Dai H, Zhou M, Pascua MR, Chen QM, Shen B. Methylation of FEN1 suppresses nearby phosphorylation and facilitates PCNA binding. Nat Chem Biol. 2010 Oct;6(10):766-73.
l	Guo Z, Zheng L, Xu H, Dai H, Zhou M, Pascua MR, Chen QM, Shen B. Methylation of FEN1 suppresses nearby phosphorylation and facilitates PCNA binding. Nat Chem Biol. 2010 Oct;6(10):766-73.
l	Guo Z, Zheng L, Xu H, Dai H, Zhou M, Pascua MR, Chen QM, Shen B. Methylation of FEN1 suppresses nearby phosphorylation and facilitates PCNA binding. Nat Chem Biol. 2010 Oct;6(10):766-73.
m	He W, Ma X, Yang X, Zhao Y, Qiu J, Hang H. A role for the arginine methylation of Rad9 in checkpoint control and cellular sensitivity to DNA damage. Nucleic Acids Res. 2011 Jun;39(11):4719-27.
m	He W, Ma X, Yang X, Zhao Y, Qiu J, Hang H. A role for the arginine methylation of Rad9 in checkpoint control and cellular sensitivity to DNA damage. Nucleic Acids Res. 2011 Jun;39(11):4719-27.
m	He W, Ma X, Yang X, Zhao Y, Qiu J, Hang H. A role for the arginine methylation of Rad9 in checkpoint control and cellular sensitivity to DNA damage. Nucleic Acids Res. 2011 Jun;39(11):4719-27.

Supplementary Figure 1. Deduction of PRMT5-dependent methylation determinant sequence. 26 PRMT5's substrates with protein sequences centered at the methylation residue R were aligned and the consensus sequence was deduced with the showing up frequency (possibility) among the 26 sequences.

Supplementary Figure 2

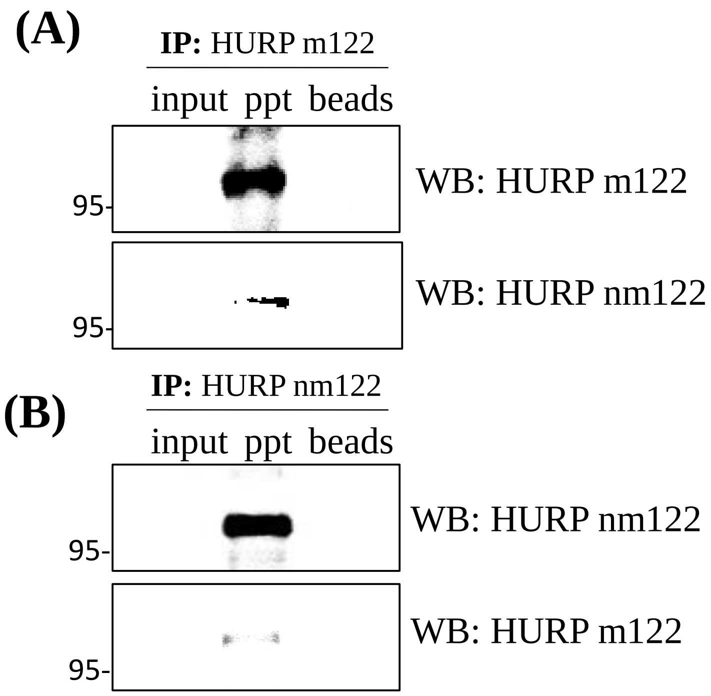

Supplementary Figure 2. The HURP m122 antibodies and nm122 antibodies almost did not cross react. Immunoprecipitations employing antibodies against HURP m122 (A) or nm122 (B) were performed, followed by Western blots using antibodies against HURP m122 or HURP nm122. Very limited amount of the immunoprecipitated HURP m122 was detected by nm122 antibodies, similarly, the immunoprecipitated nm122 almost could not be recognized by m122 antibodies.

Supplementary Figure 3

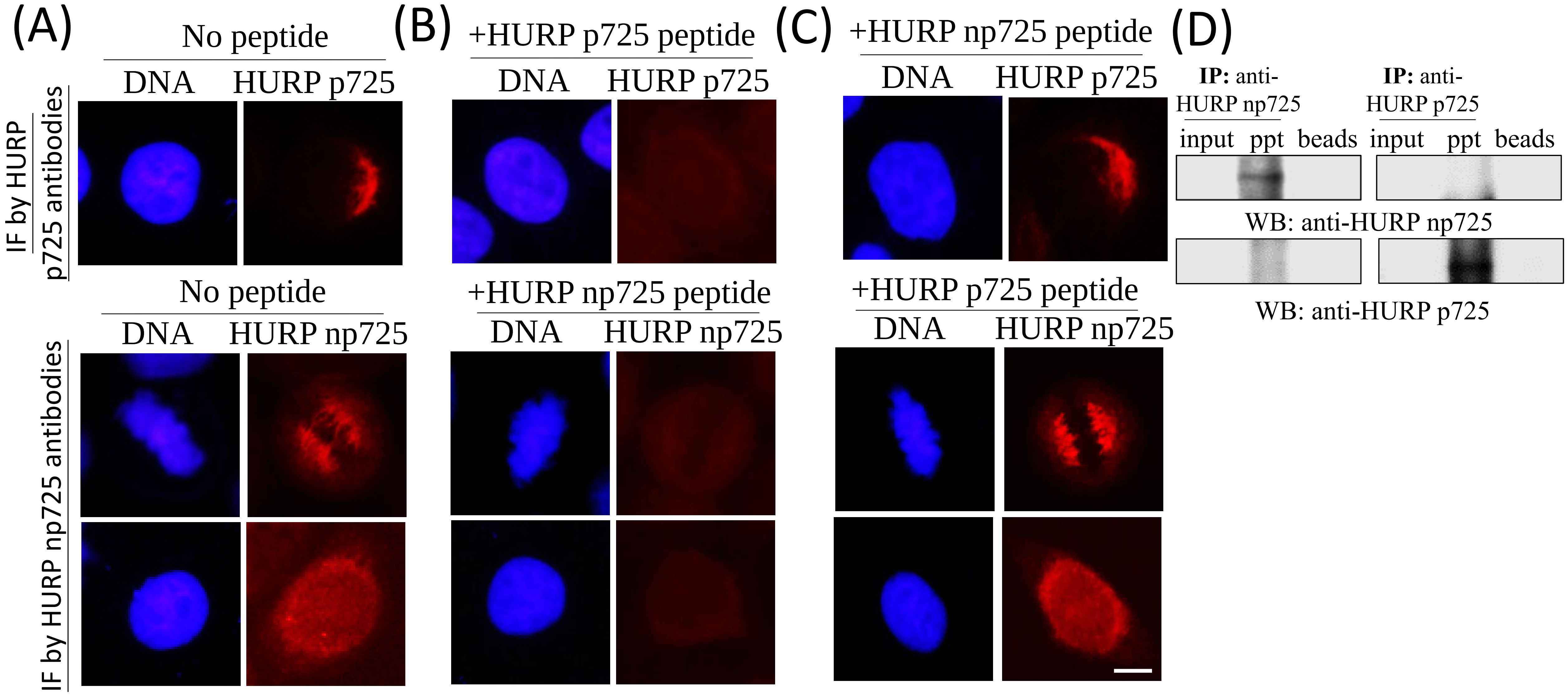

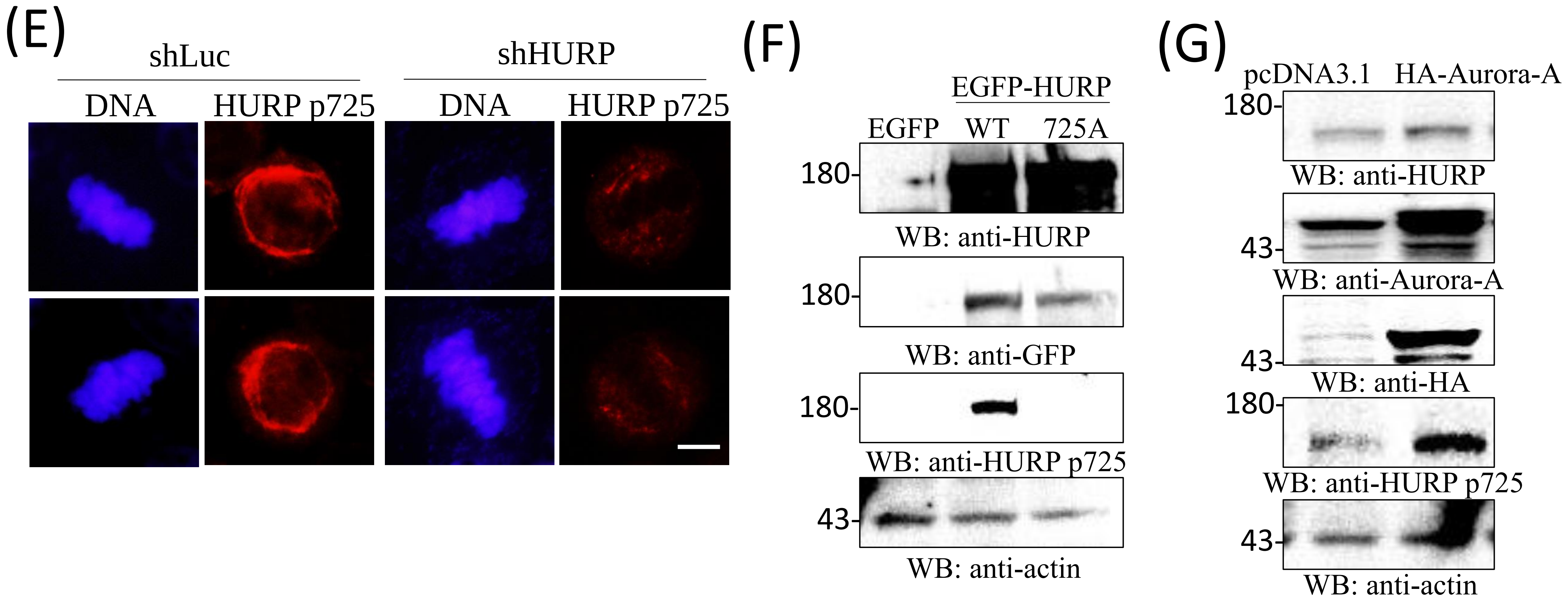
(E)
shLuc
shHURP
DNA
HURP p725
DNA
HURP p725
(F)
EGFP-HURP
EGFP
WT
725A
180-
WB: anti-HURP
180-
WB: anti-GFP
180-
WB: anti-HURP p725
43-
WB: anti-actin
(G)
pcDNA3.1
HA-Aurora-A
180-
WB: anti-HURP
43-
WB: anti-Aurora-A
43-
WB: anti-HA
180-
WB: anti-HURP p725
43-
WB: anti-actin

Supplementary Figure 3. Antibody specificity of HURP p725 and np725. **(A)** Immunofluorescence (IF) was performed adopting antibodies against HURP p725 or np725. **(B)** The antibodies against HURP p725 or np725 were incubated with corresponding antigenic peptides first, and then applied to immunofluorescence. **(C)** HURP p725 antibodies were incubated with the peptide without phosphorylation at S725. Similarly, HURP np725 antibodies were incubated with the peptide containing phosphorylation at S725. Subsequently, those antibodies were applied to immunofluorescence. **(D)** Immunoprecipitations adopting antibodies against HURP p725 or np725 were performed first, Western blot employing antibodies against HURP p725 or np725 were then followed. **(E)** Immunofluorescence adopting HURP p725 antibodies was performed in shLuc or shHURP HeLa cells. **(F)** HURP p725 antibodies could not recognize HURP 725A. HEK293 harboring EGFP-HURP WT or 725A were applied to Western blot adopting antibodies against HURP p725. **(G)** Overexpression of Aurora-A increased the level of HURP p725. HEK293 cells harboring pcDNA3.1 or HA-Aurora-A were subjected to Western blots detecting the level of HURP p725.
